# A conceptual framework for patient-centered fertility treatment

**DOI:** 10.1186/s12978-017-0375-5

**Published:** 2017-09-07

**Authors:** Elizabeth A. Duthie, Alexandra Cooper, Joseph B. Davis, Katherine D. Schoyer, Jay Sandlow, Estil Y. Strawn, Kathryn E. Flynn

**Affiliations:** 1 Center for Patient Care and Outcomes Research, Medical College of Wisconsin, 8701 Watertown Plank Rd, Milwaukee, WI 53226 USA; 2 Social Science Research Institute, Duke University, Box 90989, Durham, NC 27708 USA; 3 Reproductive Medicine Associates of New York, 635 Madison Ave, New York, NY 10022 USA; 4 Department of Urology, Medical College of Wisconsin, 9200 W Wisconsin Ave, Milwaukee, WI 53226 USA; 5 Department of Obstetrics & Gynecology, Division of Reproductive Endocrinology and Infertility, Medical College of Wisconsin, 9200 W Wisconsin Ave, Milwaukee, WI 53226 USA

**Keywords:** Patient-centered care, Infertility, Fertility, Decision making, Male, Female, Couples

## Abstract

**Background:**

Patient-centered care is a pillar of quality health care and is important to patients experiencing infertility. In this study we used empirical, in-depth data on couples’ experiences of infertility treatment decision making to inform and revise a conceptual framework for patient-centered fertility treatment that was developed based on health care professionals’ conceptualizations of fertility treatment, covering effectiveness, burden, safety, and costs.

**Methods:**

In this prospective, longitudinal mixed methods study, we collected data from both members (separately) of 37 couples who scheduled an initial consult with a reproductive specialist. Data collection occurred 1 week before the initial consultation, 1 week after the initial consultation, and then roughly 2, 4, 8, and 12 months later. Data collection included semi-structured qualitative interviews, self-reported questionnaires, and medical record review. Interviews were recorded, transcribed, and content analyzed in NVivo. A single coder analyzed all transcripts, with > 25% of transcripts coded by a second coder to ensure quality control and consistency.

**Results:**

Content analysis of the interview transcripts revealed 6 treatment dimensions: effectiveness, physical and emotional burden, time, cost, potential risks, and genetic parentage. Thus, the revised framework for patient-centered fertility treatment retains much from the original framework, with modification to one dimension (from safety to potential risks) and the addition of two dimensions (time and genetic parentage). For patients and their partners making fertility treatment decisions, tradeoffs are explicitly considered *across* dimensions as opposed to each dimension being considered on its own.

**Conclusions:**

Patient-centered fertility treatment should account for the dimensions of treatment that patients and their partners weigh when making decisions about how to add a child to their family. Based on the lived experiences of couples seeking specialist medical care for infertility, this revised conceptual framework can be used to inform patient-centered treatment and research on infertility and to develop decision support tools for patients and providers.

## Plain English summary

Couples who are experiencing infertility must weigh multiple factors when making decisions about medical testing and treatment for infertility. It is important they receive care that is “patient-centered”, that is, care that is sensitive to their preferences, needs, and values. Previously, a conceptual framework was developed based on health care professionals’ conceptualizations of fertility treatment to explain the dimensions of treatment including effectiveness, burden, safety, and costs. In this study we used data from patients and their partners to evaluate and refine this framework.

We collected data through interviews, questionnaires, and medical records from both members of 37 couples who scheduled a new appointment with a reproductive specialist. Data collection occurred 1 week before the first appointment, 1 week after the appointment, and then roughly 2, 4, 8, and 12 months later. Our careful examination of the data revealed 6 treatment dimensions: effectiveness, physical and emotional burden, time, cost, potential risks, and genetic parentage. This was similar to the framework developed with health care professionals with a few modifications. We also noted that for patients and their partners making fertility treatment decisions, tradeoffs were considered *across* dimensions as opposed to each dimension being considered on its own. These results can inform patient-centered treatment, research in infertility, and the development of decision support tools for patients and providers.

## Background

The Institute of Medicine has long prioritized patient-centeredness in care, that is, “providing care that is respectful of and responsive to individual patient preferences, needs, and values and ensuring that patient values guide all clinical decisions” [1]. However, patient-centeredness has been called a “neglected outcome” in fertility care [2]. Research from Europe has suggested that while patients from many different countries view patient-centered fertility care similarly [3], there is a gap in priorities between patients and reproductive specialists. In a discrete choice experiment in Dutch and Belgian fertility clinics, reproductive specialists overestimated how much patients value pregnancy rates and underestimated the value patients place on patient-centered care [4]. Research from Sweden has evaluated the quality of fertility care as perceived by men, women, and health care professionals [5–7]. Overall, relatively little empirical work on patient-centered care has been conducted, especially in the United States (US), where health insurance coverage of testing and treatment varies widely by state.

Decisions regarding testing and treatment for infertility can be complicated by the presence of multiple decision makers who necessarily bear different costs and may have conflicting preferences for how to resolve infertility. When couples are seeking treatment, patient-centered fertility care should balance the preferences, needs, and values of both members of the couple. While couples seeking medical treatment for infertility ostensibly do so because they strongly desire to have a child, this goal is balanced by other priorities [8], particularly maintaining a close relationship with a partner [9].

In 2014, Dancet et al. proposed a framework for patient-centered fertility treatment with four dimensions of fertility treatment that directly affect patients and influence their treatment decisions, namely effectiveness, burden, safety, and costs [10]. They placed the patient at the center of their framework, noting that too often in clinical practice and research the perspectives of patients are left unexplored or are presented as secondary to professional views. However, the authors also noted that the four treatment dimensions included in the model were derived from health care professionals’ conceptualizations of fertility treatment, rather than from patients themselves. They called for empirical research on patients’ experiences of infertility treatment decision making to revise the model while keeping patients at the center.

**Fig. 1:**
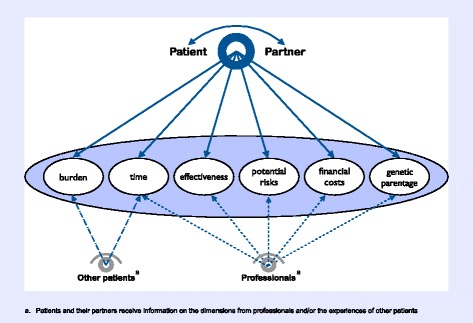
Conceptual framework for patient-centered fertility treatment

In the context of a prospective, longitudinal cohort study on US couples’ infertility decision making, we examined a subset of patients and their partners who pursued multiple medical treatments for infertility. We provide data from each couple’s decision-making process on the four dimensions outlined in the original framework from Dancet et al. (2014) [10]. We then present a revised framework based on empirical evidence from the experiences of real patients (Fig. 1).

## Methods

### Participants

We recruited new patients and their partners from an infertility practice at a large academic medical center in suburban Milwaukee, Wisconsin. Our target sample size of 35 couples was based on our goal of reaching theoretical saturation (“redundancy”) [11]. Between May and November of 2013, letters detailing the research study were mailed to new patients who scheduled first consultations with a reproductive specialist and who met preliminary criteria: (1) an address within 30 miles of the clinic; (2) a partner who had not already been contacted about the study; and (3) a first-consultation date at least 1 week in the future. Because of the short window of time to recruit to the study before the first appointment, people were only invited to participate once; no follow-up attempts were made. Potential participants who responded to the letter and who met additional inclusion criteria, including having a partner who was willing to participate, not having previously had a child using any assisted reproductive technology (ART) or any fertility treatments under the care of a reproductive specialist, comfort communicating in English, and the ability to provide baseline data before the first appointment with the reproductive specialist, were invited to join with their partner. Forty-one respondents and their partners were eligible, enrolled in the study, and provided written informed consent; 37 couples completed the year-long study (Table 1). The study was approved by the Medical College of Wisconsin/Froedtert Hospital Institutional Review Board.

**Table 1:** Sample characteristics at baseline

Descriptor, n (%)	Patients	Partners
Gender	*N* = 37	*N* = 37
Female	37 (100.0%)	2 (5.4%)
Male	0 (0%)	35 (94.6%)
Age		
≤ 30	16 (43.2%)	10 (27.0%)
31–40	18 (48.7%)	19 (51.4%)
≥ 41	3 (8.1%)	8 (21.6%)
Race		
White	32 (86.5%)	34 (91.9%)
Black or African American	2 (5.4%)	2 (5.4%)
Asian	2 (5.4%)	1 (2.7%)
Multiple	1 (2.7%)	0 (0%)
Educational Attainment		
Some High School or HS Grad/GED	3 (8.1%)	6 (16.2%)
Some College/Tech Degree/AA	3 (8.1%)	6 (16.2%)
College Degree (BA/BS)	16 (43.2%)	16 (43.2%)
Advanced Degree (MA, PhD, MD)	15 (40.5%)	9 (24.3%)
Personal Income^a^		
$39,999 or less	15 (40.5%)	10 (27.8%)
$40,000 to $59,999	11 (29.7%)	10 (27.8%)
$60,000 to $79,999	4 (10.8%)	8 (22.2%)
$80,000 or more	7 (18.9%)	8 (22.2%)
Employment Status		
Homemaker	3 (8.1%)	0 (0%)
Full-time employed	31 (83.8%)	34 (91.9)
Student	1 (2.7%)	2 (5.4%)
Employed and in school	2 (5.4%)	1 (2.7%)
Self-Reported Health		
Excellent	5 (13.5%)	8 (21.6%)
Very Good	23 (62.2%)	15 (40.5%)
Good	8 (21.6%)	11 (29.7%)
Fair	1 (2.7%)	3 (8.1%)
Health Conditions: “Ever told by a doctor you have…”		
High Blood pressure^b^	5 (13.9%)	4 (11.1%)
Diabetes^a^	1 (2.8%)	4 (10.8%)
Cancer	2 (5.4%)	1 (2.7%)
Depression	3 (8.1%)	3 (8.1%)
Anxiety	10 (27.0%)	3 (8.1%)
BMI^a^		
Underweight (< 18.5)	1 (2.8%)	2 (5.4%)
Normal weight (18.5 ≥ BMI < 25.0)	17 (47.2%)	10 (27.0%)
Overweight (25.0 ≥ BMI < 30.0)	12 (33.3%)	11 (29.7%)
Obese (≥ 30.0)	6 (16.7%)	14 (37.8%)

^a^Data unavailable for one participant

^b^Data unavailable for two participants

### Procedure

Each participant contributed data at six time points over 12 months, completing surveys, in-depth one-on-one interviews, and granting medical-record access. Each member of the participating couples was interviewed separately at: (1) 1 week prior to the first scheduled consultation with the reproductive specialist; (2) 1 week after the first consultation; (3) after receiving test results or about 2 months after the first consult if no testing was done; (4) 4 months post-consult; (5) 8 months post-consult; and (6) 12 months post-consult. Once paired with one of the two trained interviewers, participants remained with the same interviewer across all time-points. At each time-point, participants completed a self-administered questionnaire using REDCap [12] and then participated in a semi-structured interview. Couples who became pregnant or completed an adoption during the study period (and so were not currently making fertility-related treatment decisions) did not participate in interviews at the 2-month, 4-month, and 8-month time points, but they were contacted via email with a link to REDCap so they could complete surveys. All interviews were audio-recorded and transcribed verbatim.

### Analysis

Our content analysis procedure included regular meetings by 4 members of the research team to develop and revise a coding scheme. The development of our codebook and our analytic procedures for team-based coding followed recommendations by MacQueen et al. (1998) [13]. Two independent coders categorized participant responses using NVivo. We double coded 25% of transcripts. We held regular team meetings to check reliability and consistency and resolve discrepancies. The resulting data document the respondents’ lived experiences in real time before, during, and after making challenging medical decisions about how best to build their families, providing rich data to inform and revise the framework of patient-centered fertility treatment. Results are presented by each of the treatment dimensions represented in the Framework. While the analytic procedures and conceptual framework cover all couples in our sample, for this manuscript we present data from couples who experienced multiple medical treatments, including in vitro fertilization (IVF), during the study period (46 transcripts from 8 participants).

## Results

**Fig. 2:**
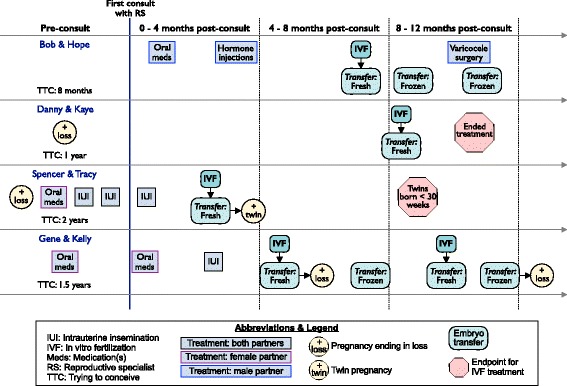
Treatment overview

Table 1 shows the demographic and health characteristics of the full sample at baseline. The four couples who completed at least one cycle of IVF within the one-year study period were similar in some ways (all non-Hispanic white, heterosexual, and married) but diverse in others, such as age (range: 24–48) and personal annual income (range: < $40,000 - ≥ $200,000). While these four couples eventually pursued IVF, each arrived at that decision via a unique treatment path and experienced different outcomes (Fig. 2). Before the initial consultation with a reproductive specialist, two couples had experienced a pregnancy and subsequent loss, two had tried oral medications alone, and one had tried intrauterine insemination (not with a reproductive specialist). At 12 months after meeting the reproductive specialist, all couples had tried multiple medical treatments including IVF. Two couples did not have any pregnancies during the 12-month period, one couple delivered premature twins, and one couple had two pregnancies with subsequent losses.

### Revised framework for patient-Centered fertility treatment

Our revised framework for patient-centered fertility treatment retains much from the original framework. We include 6 dimensions, 3 unchanged from the original, 1 modified, and 2 additional. Definitions for each dimension are presented in Table 2, with representative quotes from participants in Table 3. In what follows, we provide an overview of participants’ experiences along each dimension. It quickly becomes evident within the sections below and in many of the quotes, that for patients making treatment decisions, tradeoffs are explicitly considered *across* dimensions as opposed to each dimension being considered on its own.

**Table 2:** Dimensions of patient-centered treatment

	Definition	Includes
Effectiveness	The likelihood that a treatment will result in a desired outcome, which may be broadly understood as achieving parenthood or more narrowly construed as achieving pregnancy and live birth within a particular time-frame	Estimated pregnancy success rates for treatment; Estimated live birth rates for treatment; Estimated number of treatment cycles to achieve pregnancy
Burden	The physical and emotional workload and responsibility that a treatment requires of patients and their partners as well as “the impact of treatment on patient functioning and well-being”	Pain/discomfort of treatment; Strain on relationships; Stress and anxiety associated with treatment
Time	The amount of time involved in treatment; time to achieving parenthood	Time involved in treatments (e.g. appointments); Estimated time to parenthood for treatment; Effect of additional elapsed time on future options
Financial Costs	The out-of-pocket cost of a treatment	Price tag of treatment options; Payment plans/options; Effect of cost on future options
Potential Risks	The negative outcomes associated with treatment that may or may not actually occur	Maternal risks (e.g. OHSS); Fetal/infant/child risks (e.g. prematurity); Multiple gestation/birth
Genetic Parentage	Genetic/biological connection to child	Whether a treatment involves the use of a patient’s and partner’s own gametes, or involves donated genetic material, such as sperm, egg, or embryo

**Table 3:** Representative/illustrative quotations on each dimension

Effectiveness	I think IVF is pretty much our only option or chance. ~ Bob, 3
	I think [IVF] would be kind of stressful, very time consuming. But I’d be willing to do it if the end result was having a kid. ~ Kelly, 3
	We’ve been waiting so long. We’re both ready, so let’s do what will work the best, the fastest. ~ Gene, 1
Burden	My apprehension is this getting in the way of my job. Is this infertility, whatever we have to go through, going to affect my ability to work, my ability to function on a daily level? I clearly don’t want anyone to know. I don’t want my family to know. I don’t want my friends to know…, and having in vitro fertilization sometimes knocks you off your feet and you have to be on bed rest, and I don’t want that to get in the way of everything. ~ Hope, 1
	I looked at my husband and I said, “I can’t do it.” And he looked at me and said, “No problem.” I said, “I can’t do it again. I don’t want to do it again.” I felt so vulnerable. I don’t like it. Take me off the emotional rollercoaster ride; I’d rather be childless. I really would. ~ Kaye, 6
	This [IUI] seemed like the easiest route right now I guess. ~ Spencer, 3
Time	Obviously, we want to have a kid sooner rather than later. ~ Gene, 2
	My main goal is obviously to keep our relationship good but also push this along as fast as we can. ~ Tracy, 2
	She’s [Hope] concerned about going through IVF. What does that entail? And is it bed rest? How much time would that be? ~ Bob, 2
Financial Costs	It [IVF] is a huge time commitment. It’s a huge emotional commitment. And the financial piece just muddies the hell out of everything. It makes the decision-making process even more difficult for people. ~ Kaye, 5
	[We haven’t totally ruled anything out, but I think we’re unlikely to try] IVF only because at least from the data that my wife has, which is what she’s basing the decision on which is what I’m basing the decision on, it doesn’t look like the bang for the buck is really all that good. At my wife’s age, that much money with that low a success rate, no, that’s not – if that’s where it’s at then I think we’re both gonna take a pass on that. ~ Danny, 1
	I know he’s [Spencer] going to want to try IUI one more time, which is fine, but then again our down payment [for IVF] is going to be smaller. It’s really hard to choose. I wish I could talk with the doctor a little bit more and [ask], “If you were me and you had done this [IUI] three times, is it a waste of money to do it again?” ~ Tracy, 3
Potential Risks	I know there’s always a risk of multiple births, and that doesn’t really bother me. If we have twins, we have twins, cool. I mean, we get two babies. ~ Kelly, 3
	[With] IVF there’s increased genetic risk, there’s increase risk of ovarian hyper-stimulation syndromes. ~ Hope, 3
	I honestly don’t see any disadvantage to it [IVF] at all. It does not appear to have any kind of real side effects for the woman. My job as the guy is relatively straightforward. So there’s no issue there. It doesn’t seem like it’s an overly invasive process. It seems relatively [simple]. ~ Danny, 4
Genetic Parentage	We both agree that we would adopt, but we have agreed that we need to rule out that we cannot biologically have our own child. ~ Kaye, 1
	Advantages [of IVF] are I could be pregnant, it would be our baby. ~ Kelly, 1
	Medically, we’ve got 11 choices. Personally, we have two…. So it’s IVF or adoption. Both my wife and I don’t feel comfortable using donor eggs or donor sperm or any of that. It’s not a judgment issue. It’s just our preference. ~ Danny, 4

### Effectiveness

Of all the treatment dimensions, effectiveness was most prominently considered by participants. For the most part, all participants shared in common the same desired outcome—parenthood—and so the effectiveness of treatment options was paramount. Effectiveness was the dimension to which the others were typically compared. In particular, participants considered the higher effectiveness of IVF as a balance to its greater physical and emotional burden and cost. This was especially noticeable when considering IVF versus other medical treatments. For couples who had unsuccessfully tried or were not good candidates for oral medications alone, the decision was typically between intrauterine insemination with ovulation induction (IUI/OI) and IVF. Within our sample, female partners tended to emphasize effectiveness while male partners gave more weight to other dimensions of treatment. For one couple, proceeding with IUI/OI was viewed as the best option by the husband (Spencer) because it was “the easiest route right now,” while for his wife (Tracy) it was viewed as a “waste” of money given the lower effectiveness. Gene and Kelly also weighed IUI/OI against IVF, with Gene preferring IUI/OI because it was less burdensome and less expensive and Kelly preferring IVF despite the acknowledged increased burden because she hoped it would be more effective.

While treatment effectiveness was important to patients making decisions about their next treatment steps, patients and their partners struggled to access accurate, consistent, and personalized information on the likelihood that treatments would be successful. Even when this information was readily available, interpretation was often a challenge. For example, even participants with advanced degrees and professional research experience struggled to understand the effect of a failed cycle on the likelihood of success in subsequent cycles.

### Physical and emotional burden

The physical and emotional burdens associated with treatments were another key dimension considered in comparing different options and making decisions about medical treatments for infertility. In describing the disadvantages of different treatment options, patients and partners alike frequently referenced the rigorous demands of the IVF protocol, including frequent clinic visits, complicated medication regimens, and, most especially, the requirement that they routinely administer injections. For some respondents, these burdens contributed to decisions to delay IVF until after they had tried other “less invasive” treatments such as IUI/OI, but anticipated physical burdens did not seem to dissuade them from considering IVF entirely.

Besides acknowledging the physical burdens associated with treatment, Kaye anticipated that the intensity of IVF could also pose an emotional burden, especially in the days following embryo transfer before getting any indication of whether or not the procedure was successful. This emotional burden was often not anticipated by other participants, but they later described how they experienced burden during the IVF process. Thus the burden dimension represents both the more obvious physical burden as well as the psychosocial burden.

Anticipating the nature and degree of treatment burdens prior to attempting a treatment is a challenge. The perspectives of patients and partners on the significance of treatment burdens for their decision making can change over time as they acquire experience with treatment. In essence, physical and emotional burdens can feel more burdensome than expected, especially if they persist over multiple (unsuccessful) cycles of treatment.

### Time

The amount of time associated with treatment was also considered by patients and partners when they considered their options. Time seems closely related to effectiveness (e.g. treatments vary in terms of the average amount of time from uptake to successful outcome) and burden (e.g. treatments vary in terms of time required at the clinic for appointments), but it does not overlap completely and thus warrants inclusion as its own dimension. Aspects of time that emerged in the data included considerations about time involved in treatment and required for a given treatment to work; patients and their partners also discussed time in relation to the opportunity cost of pursuing a particular treatment and its effect on future options.

Even before meeting with the reproductive specialist, patients and their partners expressed concerns about how much time particular treatments would require within their day-to-day lives. Hope, a healthcare provider with a demanding clinical schedule, described her anxiety about the potential for a time-intensive IVF treatment protocol to add burden to her already stressful professional life, something she noted that her husband would not have to deal with.

Time was also a factor for respondents who expressed a sense of urgency in their desire to become parents, often relating time with effectiveness. Some, like Bob, Hope, Danny, and Kaye, discussed concerns that their own or their partner’s age omitted some options from likely being effective and/or imposed a timeline on their family-building efforts, pushing them to be decisive and start more intensive and expensive, yet often also more effective treatments sooner rather than later. Younger respondents were not immune from feeling urgency, describing a sense that they had been waiting so long for a baby already that their patience for options perceived as likely to take more time to work was running low. Such feelings contributed to ambivalence among some respondents who struggled to balance their acknowledged impatience with their desire to progress through treatment without “skipping” or “leapfrogging” right to IVF.

### Costs

Even prior to meeting for the first time with a reproductive specialist, patients and their partners understood that treatments vary in terms of their financial costs. At this early stage, most also knew that IVF in particular carried a hefty price tag. Despite this, some respondents anticipated that they would pursue IVF because they believed it would prove to be their best or only option to achieve parenthood. Others hoped that some less expensive treatment would prove effective and they would not need to worry about the cost of IVF. It merits emphasizing that these different perspectives were, in more than one case, jointly held by different members of a single couple who needed to reach consensus about a necessarily shared course of action.

Couples often considered the cost of treatment in light of treatment effectiveness. Respondents described uncertainty about the wisdom of choosing a more expensive treatment that would be more likely to result in the desired outcome versus choosing a less expensive treatment that was also less likely to be effective: If, for example, they tried IUI/OI and it was successful, there would be no need to try IVF and they would save tens of thousands of dollars. However, because IUI/OI was less likely to be successful, they risked spending a few thousand dollars on a failed treatment when that money could have been put toward IVF. In only one couple did neither partner express significant concerns about the cost of treatment: Bob and Hope rarely mentioned treatment costs and they were the only couple of the four to decide not to purchase a treatment package offering a substantial refund if multiple treatments were unsuccessful; they also reported the highest household income of the four couples.

Within couples, partners were not always in agreement about the degree to which cost should be considered in their decisions. For example, Kaye described feeling frustrated by her perception that she was more concerned about treatment costs than Danny. Danny, meanwhile, wanted Kaye to focus on positive thinking, believing that if she was stressed (including about the cost of treatment), treatment would be less likely to work. In other cases, participants’ perceptions of the role played by cost in their partner’s decision-making process did not necessarily match their partner’s description of their own process. For example, Tracy and Kelly both believed that cost was determinative for their husbands in deciding between IUI/OI and IVF, but neither Spencer nor Gene described their decision-making processes in that way.

### Potential risks

Throughout these nearly 50 interviews, neither patients nor partners seemed to consider potential risks (or safety, in Dancet and colleagues’ original model) as an important dimension of fertility treatment. While some respondents stated that, hypothetically, they would have serious reservations about or be unwilling to consider treatments that pose risks to their own or their partner’s health or to the health of a child conceived through that treatment, they did not associate any available treatments with this kind of risk. Indeed, at least one participant confidently stated that IVF could not put a mother or baby at risk in any way. More common was the acknowledgement that any medical procedure involves some inherent risks. Most respondents indicated some understanding that IVF can cause side effects, but these were not considered health “risks” so much as burdens that could be endured for the greater good of effective treatment.

Twin gestation, a potential outcome of fertility treatments that the ASRM, ACOG, and others regard as a maternal and fetal health risk, was perceived by some patients and partners as a net positive. While participants did express understanding that there were limits to the number of embryos/fetuses that could gestate healthily at once, most did not think twins exceeded that limit, and at least one participant felt similarly about triplets. Bob and Hope were the only couple who perceived a twin pregnancy as a risk they hoped to avoid. The potential risk of multiples did not deter them from considering any particular treatment, though when they opted to pursue IVF, they were clear about their preference to transfer only one embryo at a time.

### Genetic parentage

Family-building paths vary in terms of the genetic connection a child and parents will have. This is perhaps most obvious when adoption is a path under consideration, but it is also the case with some treatments. IUI, for example, can involve donor sperm. With IVF, a resulting child can be the genetic child of both parents (when no gametes are donated), one parent but not the other (when either sperm or eggs are donated), or neither parent (when embryos or eggs and sperm separately are donated). Even when respondents did not raise the topic of genetic parentage explicitly, subtext suggested that this treatment dimension was an important consideration for most couples. For these reasons, we have added it to the framework as a treatment dimension.

Some participants were explicit in their consideration of genetic parentage. Danny and Kaye dismissed medical treatments that involved third party genetic material. For participants who did not raise the topic independently, we asked questions about openness to donor gametes and embryos. Some were unwilling to speculate, even hypothetically, if they had no reason to believe that it would improve effectiveness of treatment in their specific case. Others described a preference for treatment with their own gametes, but to varying degrees they left the door open to donor eggs, sperm, or embryos down the road if they were not successful with their own genetic material and if donor material would improve treatment effectiveness. No respondents had a prima facie preference for donor gametes or embryos, regardless of the degree to which donated material could improve the likelihood of treatment success.

## Discussion

The in-depth interviews we conducted with patients and partners pursuing specialist treatment for infertility provided support for the framework for patient-centered fertility treatment described by Dancet et al. (2014). We propose a few modifications to the framework to improve its alignment with the lived experiences of U.S. couples seeking specialist medical care for infertility.

First, we expanded the “safety” dimension to include potential risks of all kinds. In this study, patients and their partners understood that different treatments are associated, to varying degrees, with discomfort or inconvenience, but no one considered any treatments as *unsafe*. We acknowledge the concerted efforts that reproductive specialists make using verbal, written, and visual formats to inform patients about any physical, psychological, and/or familial (including fetal, neonatal, and childhood) risks of different treatment options. However, patients may not fully understand or correctly interpret this counseling. While it is clear that safety did not constitute a concern about treatment from the viewpoint of patients, our data provide less insight on the reason for this lack of concern. Regardless, clinicians who advise patients on their treatment options must continue to provide education about the risks of treatments, with the goal that patients understand both the likelihood and the gravity of risks.

Second, where Dancet et al. left room in the framework for additional “unknown” patient-centered treatment dimensions, we have added the dimensions of “time” and “genetic parentage.” These were important considerations for all of these couples. Regarding time, some patients expressed concern about the time required to undergo treatment. More strikingly, patients and partners of all ages experienced a sense of urgency to resolve their infertility with many describing stress and anxiety due to the unexpected delays in starting their family. Older patients also referenced a biological imperative to move quickly. In both cases, this sense of urgency featured as a consideration for patients in choosing IVF. Regarding genetic parentage, clinicians who counsel patients on infertility treatment options should seek to understand the degree to which their patients’ family-building goals include parenting a child with their own (and/or their partner’s) genes. This conversation should be revisited from time-to-time as our interviews suggest that couples in the early stages of treatment often anticipate they will become more willing to pursue options including donated genetic material in the future, if treatments with their own genes fail to produce results.

In addition to these modifications, our interviews provided evidence for two points relating to the interpretation of the framework. First, it is critical that the framework be viewed with the understanding that patients weigh dimensions against one another rather than consider them in isolation. For example, two treatments which cost the same will be viewed as having different value if one is more effective or less burdensome than the other. With this in mind, when counseling patients on their treatment options, reproductive specialists should help patients to identify and weigh tradeoffs across all the treatment dimensions. As part of this conversation, clinicians should aim to understand patients’ priorities for treatment. One approach to doing so while also helping patients to clarify their own positions in relation to the dimensions is to work through the Family-Building Priorities Tool with new patients [9]. Adoption of this tool will show clinicians how patients prioritize factors associated with different treatments, giving them a sense of what patients value most as they approach treatment decisions. Such conversations could also serve as an opportunity for clinicians to highlight potential risks associated with different treatments, a dimension that patients in this study did not routinely consider in their decision making but which could have serious effects on their own health and wellbeing as well as that of their children. For example, before starting treatment, Spencer and Tracy had no health-related concerns about the possibility of multiple-gestation; their only concerns related to the cost of raising twins and potential strain on their relationship. They felt very differently when, less than 7 months later, their premature twins needed a long stay in the neonatal intensive care unit following an emergency cesarean-section. Second, the dimensions will likely be experienced and valued differently by couples depending on how experienced they are with the available treatments. Prior to starting IVF treatment, for example, couples’ concerns about the burdens of treatment are hypothetical. After one or two cycles of treatment they may have found that some aspects of treatment are less burdensome than expected while others feel intolerable. For clinicians who aim to provide patient-centered care, discussion about a couple’s goals, expectations, and limits must be understood as an ongoing process and not as a one-time event.

This research was conducted at a single, suburban academic medical center in a convenience sample of new patients. All research necessitates tradeoffs between breadth and depth, and the strength of this data is in its depth. While a small number of patients and their partners provided data, in nearly 50 encounters with both male and female partners we were able to understand in detail their treatment journeys over a 12-month period to confirm, augment, and revise a patient-centered treatment framework that was developed based on health care professionals’ conceptualizations of fertility treatment.

The revised framework covers six dimensions that can be used to inform patient-centered treatment, research in infertility, and the development of decision support tools for patients and providers. While this framework is limited to dimensions that are weighed when seeking treatment, the framework could be expanded beyond dimensions of treatment to include options for resolving infertility that do not involve medical intervention, such as expectant management, adoption, or choosing to live child-free. One participant expressed a desire for broader information than a clinic can provide about outcomes: “I would love to be able to call the clinic and say, ‘Can you tell me how many women who come in my age decide to go [with] IVF?’ That would be so powerful for me to make a more informed decision. I highly suspect there were women [aged] 41 and 42 who walked in that clinic and said, ‘You know what? I’m going to do something different.’ And what did they do? Did they go naturally? Did they adopt? I’d be really interested to know what the outcomes were for those women. And maybe they chose to be childless and they were okay with that, or maybe they chose to adopt, or maybe they actually were successful [trying on their own].”

## Conclusions

Patient-centered fertility treatment should account for the dimensions of treatment that patients and their partners must weigh when making decisions about how to add a child to their family. These include effectiveness, physical and emotional burden, time, cost, potential risks, and genetic parentage.

## Abbreviations

ART: Assisted reproductive technology
IUI/OI: Intrauterine insemination with ovulation induction
IVF: In vitro fertilization
